# The conversation matters: a qualitative study exploring the implementation of alcohol screening and brief interventions in antenatal care in Scotland

**DOI:** 10.1186/s12884-019-2431-3

**Published:** 2019-09-04

**Authors:** Lisa Schölin, Niamh Fitzgerald

**Affiliations:** 1 School of Health in Social Sciences, University of Edinburgh, Teviot Place, Edinburgh, EH8 9AG UK; 2 Institute for Social Marketing, University of Stirling, Stirling, FK9 4LA UK

**Keywords:** Alcohol, Screening and brief interventions (SBI), Antenatal care, Implementation, Pregnancy, PRISM

## Abstract

**Background:**

Alcohol screening and brief intervention (SBI) in antenatal care is internationally recommended to prevent harm caused by alcohol exposure during pregnancy. There is, however, limited understanding of how SBI is implemented within antenatal care; particularly the approach taken by midwives. This study aimed to explore the implementation of a national antenatal SBI programme in Scotland.

**Methods:**

Qualitative interviews were conducted with antenatal SBI implementation leaders (*N* = 8) in eight Scottish health boards. Interviews were analysed thematically and using the ‘practical, robust implementation and sustainability model’ (PRISM) to understand differences in implementation across health boards and perceived setting-specific barriers and challenges.

**Results:**

In several health boards, where reported maternal alcohol use was lower than expected, implementation leaders sought to optimize enquires about women’s alcohol use to facilitate honest disclosure. Strategies focused on having positive conversations, exploring pre-pregnancy drinking habits, and building a trusting relationship between pregnant women and midwives. Women’s responses were encouraging and disclosure rates appeared improved, though with some unexpected variation over time. Adapting the intervention to the local context was also considered important.

**Conclusions:**

This is the first study to explore implementation leaders’ experiences of antenatal SBI delivery and identify possible changes in disclosure rates arising from the approach taken. In contrast with current antenatal alcohol screening recommendations, a conversational approach was advocated to enhance the accuracy and honesty of reporting. This may enable provision of support to more women to prevent Fetal Alcohol Spectrum Disorders (FASD) and will therefore be of international interest.

## Background

Alcohol use in pregnancy can cause harm to the developing fetus, including growth restrictions, low birth weight, pre-term birth, and fetal alcohol spectrum disorders (FASD) [1–4]. Recent estimates suggest that 41.3% of women in the UK consume alcohol at some point during pregnancy, among the highest in the World Health Organization (WHO) European Region. Furthermore, the UK also has a high estimated Foetal Alcohol Syndrome (FAS) prevalence, which is the most severe form of FASD [5]. Identifying women who drink during pregnancy, and providing information and effective support, is therefore of public health importance.

International guidelines recommend that health professionals screen all pregnant women for alcohol use and provide an intervention to those who drink, supporting behaviour change [6]. Screening and brief intervention (SBI) consists of a short conversation focused on identifying problem drinking, motivating and facilitating reduction in drinking or abstinence to reduce the risk of harm [7]. SBI is typically intended to be delivered by a generalist health professional to patients who are not seeking treatment for alcohol problems [8]. The evidence for SBI effectiveness in reducing drinking among adults in primary care settings is relatively strong [9, 10], though not unproblematic [11] and may be less applicable to women [9]. In antenatal care, systematic reviews have cautiously supported SBI efficacy for reducing alcohol consumption during pregnancy, although few relevant studies have been published. High risk of bias and complexity of interventions contribute to important uncertainties regarding efficacy in this setting [12, 13]. Despite this evidence gap, WHO guidelines note that SBI benefits are likely to outweigh any potential adverse effects [6], justifying implementation in antenatal care.

In Scotland, clinical guidelines have highlighted antenatal care as an important setting for SBI delivery since 2003 [14]. In 2008, this was formalised as a national programme by setting a target for “alcohol brief intervention” (ABI) delivery in three priority settings, including antenatal care. ‘ABIs’ included screening, and the term can be considered synonymous with SBI [15]. A national training programme, practitioner materials, and significant funding including for specialist alcohol services accompanied the target. Official drinking guidelines for pregnant women accompanied the national SBI programme. At the time the national target was first introduced, the advice was not to “drink more than 1–2 units of alcohol once or twice per week” and not to “get drunk” [16]. This changed to an abstinence-focused message in 2010 [17]. The 14 health boards (regional health providers in the UK National Health Service) are obliged to report to the Scottish Government quarterly on their progress in delivering SBI. Within this programme, all midwives are expected to be trained, and should screen all pregnant women.

The ambitious scale, resourcing, and profile of the national SBI programme was unprecedented in the UK, comparing only with a few initiatives internationally. Evidence from the Swedish ‘Risk Drinking Project’ show that educational efforts led to improved midwife knowledge and competence in identifying pregnant women defined as having at-risk consumption patterns. However, the project had a uniform approach to addressing alcohol across primary care, occupational health care, and child and maternity care, and few details about views on implementation or adaptations have been published [18].

To date, the Scottish national SBI programme, which could provide a useful model in other jurisdictions, has not been extensively evaluated. Evaluations to date have focused on SBI delivery in primary care [19] and youth settings [20]. The aim of this paper is to explore implementation leaders’ experiences of incorporating SBI into routine practice in antenatal care under the Scottish national SBI programme. Specifically, our research question was how local health boards adapted, implemented and experienced the national SBI programme in antenatal care.

## Methods

### Study design and sample

A qualitative design was chosen to explore local health professionals’ experiences of implementing SBI in antenatal care. Participants took part in in-depth interviews, conducted by telephone to accommodate their clinical commitments, there being no good evidence of the superiority of face-to-face interviews [21]. This paper draws on secondary analysis of data from a wider study of the national SBI programme led by NF. The methods have been published in full in line with COREQ and RATS guidelines [22, 23], where they are described in detail [24].

Fourteen key people who worked as local implementation leaders were purposively recruited for the original study [25]. Sampling included leaders with experience from high-performing as well as low-performing health boards, defined as above or below the median of SBIs delivered in antenatal care. Of those sampled, one senior midwife who had initially agreed to take part was not contactable and did participate in the original study. Eight of the original 14 participants were responsible for implementation in the antenatal setting and are included in this paper: 6 of these were also responsible for SBI delivery in other settings. Participants worked as specialist midwives/nurses, Alcohol and Drug Partnership (ADP) coordinators, and a senior public health doctor with both clinical and strategic experience (Table 1). The original study obtained ethical approval from the Ethics Committee of London School of Hygiene and Tropical Medicine.

**Table 1:** Interviewee characteristics

Job/profession at time of implementation	Role (strategic and/or clinical)	Health board area	SBI performance
ADP Coordinator	Strategic	A	High
ADP Coordinator	Strategic	B	Low
Specialist Nurse (Addictions)	Clinical and strategic	C	Low
Specialist Midwife	Clinical and strategic	D	High
Specialist Nurse (Addictions)	Strategic	E	Low
Senior Medical Doctor (Public Health)	Strategic	F	Low
Specialist Midwife	Clinical and strategic	G	Low
Senior NHS Officer	Strategic	H	Low

*ADP* Alcohol and Drug Partnership

### Data collection

Identified individuals were contacted via email and invited to take part in a telephone interview. NF conducted the interviews between September and November 2013. The interviews were semi-structured and used a pre-circulated topic guide [21]. Participants were encouraged to speak freely about the SBI implementation experiences in their health board. Interviews were audio recorded and complemented by notes taken at the time of the interview. Participants verified interview notes and transcripts through member checking, with opportunity to add or clarify the interview. In accordance with ethical approval, and to reduce burden on participants from having to separately return written consent forms, all interviewees provided audio-recorded, fully-informed, formal, verbal consent and were reassured of confidentiality.

### Data analysis

All interview transcripts from the full study were reviewed for data relating to antenatal care, resulting in a final dataset of implementation leaders from eight of the eleven Scottish mainland health boards. These interviews lasted an average of 74 min each. These transcripts were subject to a detailed analysis. Both authors read the eight relevant interview transcripts several times to gain familiarity with the data. LS undertook initial inductive coding, which was discussed, and the codes were organised thematically, by NF and LS [26], the practical robust implementation and sustainability model (PRISM) was also used to organise the findings. The PRISM analysis focused on conceptualising implementation in relation to the recipients, intervention, organizational factors and external context [27].

## Results

Approaches and strategies taken to implement SBI in antenatal care varied between health boards. Table 2 outlines implementation by the four PRISM areas (recipients, program, infrastructure and sustainability, and external environment). Local structures and factors, midwives’ attitudes towards women's drinking habits in and outwith pregnancy caseloads, and time available for training were important factors for implementation. The following sections describe implementation in relation to integration into routine practice, perspectives on screening, contextual factors, and perceived outcomes. Two case studies are included to illustrate the reported impact of using different approaches to asking pregnant women about alcohol consumption.

**Table 2:** Findings organised by PRISM domains

Health board area	PRISM domain			
	Recipients	Program (intervention)	Implementation infrastructure and sustainability	External environment
A	• Difficult to arrange training due to midwives’ workloads	• TWEAK used as screening tool, as midwives were comfortable with it – needed support on how to develop the system around it • BI delivered for positive screen, referral for “higher levels of drinking”	• High performing SBI delivery^a^ • SBI delivery and reporting worked well • Antenatal perceived as an easier place to deliver SBIs – pregnant women have an appointment	• Growing knowledge of FAS facilitated implementation as midwives perceived SBIs as a good preventive strategy
B	• Midwives believed women who already have a problem would be known, others would say they do not drink • No “buy-in” from senior management		• Low performing SBI delivery^a^	• Alcohol competed with other risk factors –not joined up
C	• A lot of information leaflets were handed out – some work was being done to inform about risks • The relationship and links between implementation lead and antenatal and alcohol liaison services and antenatal were not strong • Support from Head of Midwifery, some lead midwives felt it was added work • ALNs observed that midwives did not have problems asking the question	• No agreement to include new screening instrument – used SWHMR as TWEAK was “too much” • Pathway was accepted, but adopting and recording was difficult • Pathways: i) BI and leaflet if women reported any alcohol use; ii) > 2 units per week, ≥1 score on CAGE, or alcohol or drug misuse in last 12 months by woman or partner women were referred to specialist services • All women being asked, < 1% reported drinking which led to: i) looking at how the question was asked, and ii) if information could target non-pregnant women	• Low performing SBI delivery^a^ • Incorporating into IT system facilitated recording. Initially poor uptake – made the question mandatory. • Implementation in antenatal not as successful as in A&E	• Drinking culture and hazardous alcohol use among women in general suggested < 1% reporting drinking in pregnancy was not true • The GIRFEC and Early Years Collaborative agendas directed maternity services’ work– felt SBIs needed to link up better and better links with ALNs is needed
D	• Support from Head of Midwifery, work was led forward by three midwives with free reign to implement • The programme was seen as supporting existing practice • Midwives became comfortable with asking question and referring, but found it difficult to assess when to involve social services • Apart from a few strong characters, general good receptiveness – main point to raise awareness of why it is important	• Alcohol was already part of SWHMR –the HEAT target more about how to ask the question and how to best record it • Developed new screening tool adapted from FAST, to fit the “local language”, including pre-pregnancy drinking and encouraged midwives to focus on the conversation about how and when alcohol was consumed (see Case Study 1 in Table 3) • SBIs recorded if woman had drunk since conception to address behaviour change also for unintended exposure	• High performing SBI delivery^a^ • HEAT target provided structure to the setup and emphasized that it was a governmental priority • Piloting and tweaking with a small number of midwives key to get screening tool and pathway right	• Local culture and knowledge of the local population part of developing the system • ADP funding was essential to get the work “off the ground”
E	• All midwives were trained through the national training programme • Trained each local team • Generally midwives were supportive	• SWHMR, but the alcohol questions were considered unsuitable for SBIs and were therefore adapted • Following screening; BI or referral to services • Question was repeated at 32 weeks and discussed throughout with women reporting drinking	• Low performing SBI delivery^a^	
F	• Midwives supported complete abstinence; NHS information at the time said limit to 1–2 units once or twice per week • Senior midwives were signed up for trainings but releasing frontline staff was difficult • Budget did not allow covering backfill in practices	• TWEAK was chosen as suitable screening tool • Poor coverage of routine screening • BIs were offered based on any alcohol use, in line with midwives’ views rather than positive screen	• Low performing SBI delivery^a^	• The public health agenda for midwives was perceived as too big and booking appointments long and information dense • No linking between agendas or acknowledgement of cross-over skills to address these issues • Conflicting messages of lower drinking limits influenced discussion on how to deliver SBIs
G	• Training was not adapted for maternity, took time tweak the materials • Managers were supportive to get staff trained quickly • Maternity managers gave “free reign” with input from ADP and SBI trainers	• Added screening and SBI delivery onto existing checklist • Used SWHMR (see Case Study 2 in Table 3) –FAST seen as inappropriate– and added whether woman been given information about risks • SBIs were delivered if a woman had consumed alcohol since conception, or drank ≤14 units or regular binge drank before getting pregnant	• Low performing SBI delivery^a^	• Conflicting messages with lower drinking limits influenced discussion on how to deliver SBIs • ADP supported financially to cover training costs
H	• Employed a person dedicated to deliver the SBI training	• Lack of scoping nationally into the feasibility of recording on existing systems • Felt it was more important to talk to women before they get pregnant	• Low performing SBI delivery^a^ • Midwifes felt uncomfortable asking about alcohol because it might jeopardize their relationship with women	• Other national work around recorded information about pregnancy and maternal health was not linked up with SBIs – missed opportunity

*A&E* Accident and Emergency, *SBI* Screening and Brief Intervention, *ALN* Alcohol Liaison Nurse, *CAGE* Cut down, Annoyed, Guilt, Eye-opener, *GIRFEC* Getting It Right for Every Child, *SWHMR* Scottish Women’s Handheld Maternity Record, *TWEAK* Tolerance, Worried, Eye-opener, Amnesia, Cut down

^a^ Performance ranking refers to the ranking at the time of the interview; high = above median of overall SBIs delivered in antenatal care, low = below the median

### Integrating SBI into routine practice

Participants noted the importance of senior management support in the implementation process, but this was not always available. For example, in Health Board B, there was “*no buy-in from senior people in antenatal*”. On the other hand, strong support from Head of Midwifery was instrumental in progressing the programme in several health boards.

It is true that I was starting from a lower base in terms of relationships. I didn’t have a strong link into antenatal settings [ … ] but there were good links with Head of Midwifery and it was made clear to midwives they had to do it. We needed to use that strategy to influence delivery in maternity so it was more of a top-down approach (Health Board C)

In order to report to Scottish Government, implementation included a focus on recording, integrating screening questions into existing electronic patient record systems, or (in one case) development of a new paper-based system. This influenced the construction of protocols for delivering SBI and referral to specialist services (see Table 2). More fundamentally, however, participants had to determine the intervention target group following the screening process. This was clear in Health Board F, where it was evident that midwives own attitudes did not match up with guidance at the time in a pamphlet given to women, where guidance was to limit intake to one to two units once or twice per week.

A lot of time (was) spent debating about exactly which women would we actually be delivering a BI [brief intervention] to, would it be women who were drinking above the 14 units limit [ … ] Was it women drinking more than the ‘Ready Steady Baby’ limits? Or was it actually what midwives felt strongly professionally, which was women who were actually drinking any alcohol at all in pregnancy? (Health Board F)

Two participants described how midwives’ attitudes towards pregnant women’s alcohol use influenced how SBI was implemented and designed locally. Whilst midwives were in agreement about total abstinence during pregnancy, the national guidance and training materials did not include a clear abstinence message, in contrast to the abstinence-focused approach taken in most of the local areas.

The national packs were useful after we had done the training for trainers but on the back of the work that was done, the initial antenatal packs said that women didn’t need an ABI [alcohol brief intervention] if drinking small amounts, but now anybody drinking in-pregnancy gets an ABI (Health Board D)

### Screening in the antenatal setting

There was no consensus on the best way to identify pregnant women who were drinking alcohol. Perceived feasibility, including required time, influenced the screening tool used or approach taken. For example, the TWEAK test [28] was used in two health boards, but was regarded too time consuming in another health board.

People felt that TWEAK was too much and they were trying to incorporate it into the initial booking appointment where there are a lot of questions to work through in an hour. So we thought the simplest thing to do was to stick with questions that were already there in the SWHMR [Scottish Women’s Handheld Maternity Record] (Health Board C)

Several other health boards also decided to limit change in current practice by using questions from existing standard forms. In health boards where standardized tools were used, considerations to their application to the local context was considered important (see Table 2).

In several areas, reported alcohol use elicited through standard questions was lower than expected. Implementation leaders’ knowledge of local drinking culture led them to conclude that drinking levels being reported in pregnancy were not accurate.

When you look at the [local] culture of drinking and hazardous drinking among women and in the population in general, we don’t think that less than 1% of women are drinking in pregnancy (Health Board C)

In the two case study areas (Table 3), discrepancies led to consideration of how to approach screening. It was clear that implementation leaders felt that screening questions had to flexible and not simply asked verbatim of each woman. In Case Study 1, focusing on the context of alcohol consumption was considered an effective strategy to improve reporting levels, and influenced disclosure rates in some cases. This was seen as critical for offering help to women who might benefit from cutting down, reducing the risk to the fetus in the current, and potentially future, pregnancies. Ensuring that midwives and pregnant women were comfortable with alcohol questions was important and meant adapting questions to local (not formal) language. In both case studies, additional prompts and questions to encourage trust and overcome defensive responses were key. Emphasising pre-pregnancy drinking was a strategy to identify, and therefore provide effective support to those who might benefit, which was also used by other participants.

We had a lot of discussion about it being more important to ask about alcohol consumption before pregnancy, because pregnant women are less likely to disclose when they are drinking in pregnancy because they know they are not supposed to (Health Board H)

Case Study 2 indicated an increase in reported pre-pregnancy abstinence over time, felt likely due to a change in the accuracy and honesty of women’s reporting, rather than a genuine fall in consumption. One interpretation was that women were ‘coming prepared’ to answer the questions. Another was that a recent focus by midwives on asking about parenting capacity and home circumstances may have made women fearful about disclosing heavy drinking (see Case Study 2). In this case, midwives were encouraged to probe further if women reported no alcohol use pre-pregnancy, resulted in higher levels of disclosure.

**Table 3:** Case studies from local areas

Title	Health Board	Case study text
Case study 1: a conversational approach to screening	*Area D*	*We designed a new screening tool because we felt that some of the tools for the antenatal session weren’t [in the kind of] language [used locally], midwives fed back that they weren’t comfortable with that.* *Initially we looked at how we approached the alcohol questions. We found that women tell us that they don’t drink, they will always say they don’t drink, but we know that is not true. So we had to look at a way that it was more of a conversation than about asking women about normal drinking behaviour. We asked the midwives not to ask about units, [but instead] ask when they drink, how often, what they drink, how much … so asking a young girl what her normal pattern was of drinking, she said ‘at weekends’, I asked when that started, what she drank in the house before going out, when she goes out. It’s about knowing about normal patterns of drinking, it was asking specific questions rather than asking how much you would drink. At the time I found out that she was drinking over 100 units at the weekend but initially she said she was only drinking socially at the weekend …* [emphasis added] *I think what I tended to find was that women were very defensive. They say I’m not going to drink and I’m going to stop now and it’s about reassuring them that that’s great. But also asking if it’s okay if we discuss that a bit more. Saying that we know that sometimes there are special occasions and they might say ‘I plan to have x, y, z’ or ‘I drank in my last pregnancy’ and the child is OK, then that’s more of an opening. But I would say those women are the ones that are less likely to want to have the conversation. I would ask them then about the effects of drinking to find out their knowledge then ask their permission ‘can we move on?’ and discuss other parts. Talk about their normal behaviour and ask ‘how easy is it going to be to make a change from that? How are you going to manage? What will you do to make that change?’ …* *I think the big thing for us is the local culture and the local language that we use. I was trying to get away from the midwives using the initial screening tool as a parrot fashion and questions. I felt that the problem was that people don’t want to talk about it, taboo around asking questions about alcohol. We had an FAS event day locally and one speaker put up some research saying that women are drinking 2 units and so they don’t drink what we think they are, using the initial screening tool we were finding they were drinking 2 units or 1 unit less than once a month. But from what we see locally and especially the post I do, people tell me that they know somebody that drank in pregnancy. So we knew that those figures weren’t right. So [we thought about] what do we do to get the correct information?...* *It’s also about reassuring them that they’re not being judged or there’s going to be some form of social work input. It’s about putting them at ease and having a different kind of conversation that was beneficial for us.*
Case study 2: addressing changes in reporting over time	Area G	Screening focused on current, previous and pre-pregnancy drinking. *“Obviously the generic training we got was using the FAST [Fast Alcohol Screening Test] screening tool and it was quite clear from the word go that it wasn’t appropriate when we were going down the route of abstinence in pregnancy. That was a big issue for us initially, was the abstinence message, when there was the mixed message still going on about whether it was safe enough to use the 1–2 units once or twice a week or should we go the abstinence. However we got a lot of support locally, through the ADP and through our consultants and obstetricians as well, we were very supported in the abstinence message in [our health board]. So we decided to go with the direct questions that were already existing in the SWHMR notes, of how many units of alcohol are you drinking in the pre-pregnancy and how many units of alcohol you were drinking currently, but elaborating by asking about their pattern of drinking and establishing how many units they were drinking on their heaviest drinking day to capture the binge culture.”* Over a 9 month period, disclosures of pre-pregnancy drinking fell by over 20%. *“One of the key things that we’ve found in [this health board] bearing in mind that we’ve been screening [for almost 5 years]. Over the last 6–9 months, we were noticing when our pre-pregnancy drinking data was coming in that we were actually seeing a great increase in the number of people who were actually saying that they weren’t drinking any alcohol at all outwith pregnancy, even in their pre-pregnant drinking, it was around about 50–52% throughout all localities that women were now saying that they weren’t drinking any alcohol at all...We’ve only seen within the last 6–9 months that we are finding that 50% on average are saying they don’t drink any alcohol, prior to that, when we introduced the training and the screening … you would probably be sitting at over 75% who were describing their pattern of pre-pregnancy drinking. They were quite happy to describe their pre-pregnant drinking...”* This fall was not thought to reflect an actual fall in drinking. “*We know within [this area] that we do have a problem with problematic binge drinking … we know that it’s an ongoing social factor here, so actually to look at the stats coming through of young women of childbearing age saying that they actually didn’t drink at all was questionable. So we approached the community midwives and got a feeling of their perception. We were a bit worried that it was the midwives who were losing the agenda, now that they were taking on other stuff, the GIRFEC agenda and other things going on, had this priority dropped?”* Women were thought to be ‘coming prepared’ to say they didn’t drink. “*So we approached midwives, and obviously working within this field for years, I know midwives who are really good at specific agendas and really good at their screening, and even midwives like that were actually coming forward to myself and saying “its actually surprising ourselves, we feel the message is out there now [the screening] has actually been embedded for several years that women actually come prepared to say that they don’t drink any more.”* A greater focus on parenting and home circumstances may have contributed to the change. *“There was no kind of follow on about why they weren’t admitting it. I think there has been such a big shift with the GIRFEC agenda [a national early years child wellbeing initiative] and the total booking appointment and how many questions – how in-depth midwifes now go in their whole circumstances, whereas before we did the key screening on things, like domestic abuse, we now look really into their whole lifestyle, where they’re living, what benefits they’re getting, what their partners do, if there has been any criminal past – we actually go very, very in-depth on their parenting capacity and any concerns that might rise from that now, so I don’t know whether with us going into this agenda, that the actual fear of actually admitting that they were drinking regular and there had maybe been instances linked to that, that they had maybe been a bit afraid to disclose that. I am not really sure where the reasons come behind that but certainly that’s what the feedback from a lot of the community midwives was, was that women were coming pre-prepared and weren’t openly discussing what they were previously drinking.”* What was done in response to the fall in disclosure? “*What we’ve actually done in relation to the early years collaborative/PDSA [Plan-Do-Study-Act] cycle that’s going on nationally we decided to do a bit of work within that to get a clear picture what percentages of women were advising that they don’t drink alcohol at all [pre-pregnancy] and also doing a wee bit of training for the midwifes in the community again.* *So we looked at numbers for three locality areas, so on how many women were advising that they don’t drink any alcohol at all, and it was true the numbers that came in were 49/52/50% for women drinking no alcohol pre-pregnancy. So it was reflective of the figures we were finding in [part of the health board] and what the community midwives were saying to us as well.”* Focusing on more prompts led to greater disclosure back to original levels. “*So next step was to focus on one community locality, and arranged to go out to speak to community midwives to have a conversation on what were their views on these stats over that last months. And they all replicated what has already been said that they felt women were already prepared and that they’ve been surprised about how many women … they were quite adamant that it wasn’t the competing agenda that was putting the priority down on their workload. So I then did a refresher course on what our policy is on the screening and when a brief intervention should be delivered and questioning their pattern of drinking when they’re pre-pregnant as well. And a lot of the midwives were again replying that women are telling us that they are not drinking.* *So I encouraged them to take the probing a wee bit further and say, ‘you’re obviously saying that you’ve never drank pre-pregnancy but have you ever drank before?’ and ‘what was your pattern of drinking then?’ and ‘when did you last drink?’. So that we’re kind of taking it that next step. So the results from that was that our screening on the pre pregnant jumped back up to 74% in that area. Now in the stage where I’m linking in with the team leaders in the other localities and they will feed this info back to their community midwives highlighting this need for further probing.”*

### Contextual factors affecting implementation

Wider maternal health and antenatal care policy agendas were important for success in implementing SBI. Several respondents mentioned that a focus on alcohol fitted with broader national efforts around early interventions for child wellbeing. This included the Getting It Right For Every Child (GIRFEC) agenda, aimed at improving health and wellbeing for children and young people in Scotland through timely support [29]. Participants however highlighted that the SBI programme did not necessarily align with GIRFEC or other relevant lifestyle and health agendas.

There were lots of different health improvement people going to the same target staff about different things to do with [how they address] lifestyle change etc. All these different approaches are being made to midwives and practice nurses or whatever separately – it’s not joined up (Health Board B)

Participants noted that this led to duplication of training, as addressing other lifestyle issues require similar skills.

There was a concern that there was not really a joined-up-ness about all of this. That people were being asked to be trained for talking about breastfeeding, looking for issues of domestic abuse, issues of smoking and behaviour change, and alcohol, but where was the joined up bit about it? Where could we capitalise on the shared skills, the crossover skills? (Health Board F)

A joined-up approach was pertinent considering that training midwives was a major task in many areas, requiring annual training of new trainee midwives and staff. Further, the number of health behaviours to cover in booking appointments was seen as increasing midwives’ workload and a burden for women. This appeared to create some resistance.

All these things were coming at the same time and setting an agenda that for midwives, and frankly for women coming for booking, was becoming too huge (Health Board F)

### Perceived outcomes of the SBI programme

Participants perceived that introducing SBIs had positive outcomes, including consistency in asking all women about alcohol, increased FASD awareness among pregnant women, and reinforcing existing midwife practice through improved guidance on facilitating the conversation. Screening rates were however low in many health boards, meaning midwives delivered few SBIs. Even where there were higher screening rates, the reported prevalence of drinking in pregnancy was often low. Several participants reported that midwives believed they would already know of a woman’s drinking problem and in at least one area, the implementation programme failed to overcome this reservation.

Midwives were not particularly happy with it, their reservation was that if somebody had a significant problem they would already be known and if they didn’t have that level of problem but were drinking, they were unlikely to tell you, the others who were happy to talk about it had already reduced or stopped drinking anyway … we had to accept what they were saying. All we could do was offer more follow-up support and refresher training, which no-one accepted (Health Board B)

## Discussion

This study explored the implementation of a national SBI programme in antenatal care in Scotland. We found number of barriers and facilitators to implementation, echoing previous research showing that open discussions are impeded by the topic’s sensitive nature [30], lack of an established relationship at booking [31], fear of judgement [32], and fear of child protection issues and involvement of social services [33]. Implementation leaders used several strategies to facilitate honest disclosures including positive conversations, exploring pre-pregnancy drinking habits, and building a trusting relationship between pregnant women and midwives. Women’s responses were encouraging and disclosure rates appeared improved, though with some unexpected variation over time. These findings can inform future SBI programmes.

The national SBI programme guidance suggested screening all pregnant women using a validated screening tool [34], which the WHO also recommends [6]. Formal screening instruments can facilitate discussion about alcohol [35]. For example, midwives in Sweden used the Alcohol Use Disorders Identification Test (AUDIT) as a “pedagogical tool” where conversations about current drinking were built on screening of pre-pregnancy consumption levels [18]. Our study found no universal adoption of a validated screening tool across health boards, as many adapted instruments to fit the local context. O’Brien [36] argued that universal application of SBI in antenatal care should be informed by evidence, but guidance should not specify a particular screening tool. Similarly, a recent literature review recommended development of national standards to facilitate SBI implementation, but made no recommendation on a specific screening tool best suited for maternal health services [37].

Several health boards emphasised the importance of a positive conversation and asking questions in a locally appropriate language: in a study from Norway, 61% of midwives reported that they would rather have a conversation with expectant parents without using a screening tool [38]. Furthermore an emphasis on building trust led to discussions of pre-pregnancy drinking behaviour, which was felt to be less stigmatised, and has been found to be an acceptable strategy [39]. Evidence that pre-pregnancy drinking levels predict continued alcohol use in pregnancy [40, 41] supports this approach.

Whilst screening adaptations appear to facilitate implementation, adaptation raises other questions: in one observational study in primary care, sensitivity was lost when health professionals made adaptations to prescribed screening tools [42]. Our case studies show that implementation leaders mandated a flexible approach, in order to build trust, and reported that it led to more frequent and/or more complete disclosures of alcohol consumption. The validity of informal adapted approaches merits further research, but it is also worth considering whether a more flexible approach may be valuable in other settings. McCambridge and Rollnick [43] argue for a more ‘patient-centred’ approach in primary care, to “encourage people with alcohol problems to tell us what their problems are, so that help can be provided to think these through”, and suggest this would distinguish face-to-face interventions from the simple, rigid, screening most commonly provided in electronic SBI.

One way of exploring the validity of flexible screening approaches is using biomarkers, which have been extensively studied in pregnancy, however evidence is insufficient to recommend routine use of currently available markers [44]. Recruitment bias and the lack of a gold standard reference test for in-pregnancy drinking impedes research in this area. With FASD being a leading preventable developmental deficit, innovative research and practice approaches are urgently needed to identify those who might benefit from support [45]. Combined self-report and biomarker methods have been utilised for identifying smoking in pregnancy [46]. For alcohol, cohort studies following up children’s outcomes after birth and through childhood, following biomarker testing combined with self-report screening in pregnancy, could provide further data to identify those most at risk in future [47]. Ideally, such screening would facilitate personalised feedback to women about the risk to their baby; a component of SBIs that appears to be important for changing behaviour [48].

This is the first indication that reporting rates of alcohol consumption in pregnant women may change over time, or be affected by other developments in the conversation before or after the alcohol questions are asked. Whilst Scotland-specific, the findings raise an important possibility that reduced disclosure of alcohol consumption may be an unintended consequence of a greater focus on child wellbeing/parenting readiness. This is likely to be relevant elsewhere. Current recommendations for implementing SBI by nurses and midwives tend to focus on structural and practical issues [37], with little discussion around the impact of contextual factors such as other policy agendas.

Our findings suggest that antenatal care may be a particularly sensitive ‘complex system’ in which interventions are influenced by policy agendas [49], and with feedback loops where over time women may be ‘prepared’ to answer in a certain way. Systems-informed evaluations of interventions in this setting that include consideration of unintended consequences are therefore vital [50]. Such evaluation should also consider whether an integrated approach to addressing alcohol and other public health topics in antenatal care could have helped, for example, by addressing cross-over skills, acknowledging the need to prioritise available time, ensuring that sensitive topics are not avoided, and addressing burden on staff. Finally, the drinking guidelines for pregnant women that existed at the time of implementation caused discomfort amongst midwives, who believed they should advise total abstinence. Several health boards therefore decided to offer SBI to any woman drinking in pregnancy rather than setting cut-off points for current drinking, an approach that was later reflected in the national programme. Implementation of a national programme therefore also needs to consider midwives’ own attitudes.

### Strengths and limitations

This is the first study to explore in detail the experiences of SBI implementation leaders of a large-scale primary prevention programme to prevent harm caused by alcohol exposure during pregnancy in the UK. It adds to understanding of the detailed practical and ethical dilemmas involved in establishing alcohol SBI in the antenatal setting, and is likely to be relevant to other countries. Eight of the eleven mainland health boards in Scotland were included, providing an insight into the implementation process in a majority of areas. However, views in remaining health boards and island boards may differ, as may experiences in other countries, where local research would be valuable.

## Conclusions

National resources, funding, and support from strategic, frontline and management staff were important for the implementation of SBI in antenatal care. A flexible, conversational approach to discussing alcohol with pregnant women was considered superior to formal tools, for identifying who might benefit from intervention. The approaches suggested could be implemented internationally and merit further study. Furthermore, national programmes should consider an integrated approach to health promotion in pregnancy in future, whilst recognising the potential for unintended consequences.

## Data Availability

Participants were not asked to give consent for interview transcripts to be shared, due to the small number of individuals in these roles in Scotland. The detail the full transcripts provide about local delivery of SBIs would render it easy to identify the participants. Interview schedules are available upon request from the corresponding author.

## Abbreviations

A&E: Accident and Emergency
ABI: Alcohol Brief Intervention, synonymous with SBI
ALN: Alcohol Liaison Nurse
CAGE: Cut down, Annoyed, Guilt, Eye-opener
FAS: Fetal Alcohol Syndrome
FASD: Fetal Alcohol Spectrum Disorders
FAST: Fast Alcohol Screening Test
GIRFEC: Getting It Right for Every Child
SBI: Screening and Brief Intervention, synonymous with ABI
SWHMR: Scottish Women’s Handheld Maternity Record
TWEAK: Tolerance, Worried, Eye-opener, Amnesia, Cut down
WHO: World Health Organization
